# Challenges in controlling the Ebola outbreak in two prefectures in Guinea: why did communities continue to resist?

**DOI:** 10.11694/pamj.supp.2015.22.1.6626

**Published:** 2015-10-11

**Authors:** Sylla Thiam, Alexandre Delamou, Soriba Camara, Jane Carter, Eugene Kaman Lama, Bara Ndiaye, Josephat Nyagero, John Nduba, Mor Ngom

**Affiliations:** 1 Amref Health Africa Headquarters, International Training Centre, P O Box 27691-00506, Nairobi, Kenya; 2 Centre de Formation et de Recherche de Maferinyah, Forecariah, Guinea; 3 Ecole de Santé Publique, Université Libre de Bruxelles, Bruxelles, Belgique; 4 Amref Health Africa, Conakry, Guinea; 5 Amref Health Africa West Africa Regional Office, Dakar, Senegal

**Keywords:** Ebola outbreak, community resistance, communication, community perception

## Abstract

**Introduction:**

The Ebola outbreak emerged in a remote corner of Guinea in December 2013, and spread into Liberia and Sierra Leone in the context of weak health systems. In this paper, we report on the main challenges faced by frontline health services and by communities including their perceptions and views on the current Ebola response in the Prefectures of Coyah and Forecariah in Guinea.

**Methods:**

A cross-sectional study was conducted in December 2014 using mixed approaches: (i) Desk review; (ii) Interviews; and (iii) Direct observation.

**Results:**

Almost one year after the beginning of the Ebola virus disease outbreak in West Africa, the perceptions of stakeholders and the observed reality were that the level of preparedness in the two health districts was low. The study identified poor coordination mechanisms, inadequate training of human resources and lack of equipment and supplies to field teams and health facilities as key elements that affected the response. The situation was worsened by the inadequate communication strategy, misconceptions around the disease, ignorance of local culture and customs and lack of involvement of local communities in the control strategies, within the context of poor socioeconomic development. As a result distrust developed between communities and those seeking to control the epidemic and largely contributed to the reluctance of the communities to participate and contribute to the effort.

**Conclusion:**

There is a need to rethink the way disease control interventions in the context of an emergency such as Ebola virus disease are designed, planned and implemented in low income countries.

## Introduction

The Ebola Virus Disease (EVD) outbreak emerged in a remote corner of Guinea in December 2013, and spread into Liberia and Sierra Leone in the context of weak health systems in all three countries [1, 2]. To date, around ten thousand people have died from the worst outbreak of the disease on record. At the end of November 2014, case incidence was still increasing in Guinea and Sierra Leone while stabilising or declining in Liberia. In Guinea, 75 to 148 confirmed cases were being reported on a weekly basis between October and end of November. By 1st December 17 districts out of 38 had been affected by the outbreak and more new cases reported in November with an increase of 36% compared to October 2014. In total 2,164 Ebola cases and 1,327 deaths were notified by the Ministry of Health in the beginning of December 2014 [3, 4].

In most of the affected areas interventions like case contact tracing and follow-up, referrals to a health centre when a case is suspected, safe burial practices and disease surveillance at community level were not routinely carried out. There was an insufficient number of case management centres despite the daily increasing demand. The already fragile National Health System in Guinea was rapidly overwhelmed leading to the inevitable accelerated spread of Ebola [1, 2, 5].

The situation required the implementation of immediate and vigorous additional interventions to stop the spread of the epidemic. International aid and support increased and many organizations and institutions joined the response with several projects implemented on the ground. Despite this, the disease continued to spread rapidly in the country. Moreover reports from the media and staff on the ground stressed community resistance and fear. There were individuals with symptoms refusing to seek care in the formal health system and instead resorting to home treatments from local pharmacies or traditional healers. With fear, despair and death all around, the community reluctance moved to an extreme level with riots and killings which started in April 2014, less than one month after the official confirmation of the epidemic. By September, eight people, including journalists, Ebola-related educators and health technicians had been killed in Guinea [1]. By early December community resistance had been reported in 9 of the 17 affected districts [6].

Many experts have attempted to explain the reasons behind the community resistance but most analysis was based on comments, testimonies and observations [1, 5]. Community members themselves were rarely consulted to understand their fears and perceptions for appropriate and urgent actions to be taken.

It is widely acknowledged that understanding and addressing communities’ needs and working side by side with local populations can significantly impact disease burden in affected communities. To support the efforts of the government and other institutions and organisations engaged in the fight against the expanding Ebola virus epidemic in Guinea, and to define appropriate strategic measures as part of a long term programme of health system strengthening, Amref Health Africa initiated a rapid assessment in selected sites at the peripheral level of the health system in their supported communities to identify gaps in Ebola response actions and understand the root causes for failure. This paper reports the main challenges faced by frontline health services and communities, and explores the perceptions and views on the current Ebola response in the Prefectures of Coyah and Forecariah in Guinea.

## Methods

**Study site and population**: The Prefectures of Coyah and Forecariah are located in Kindia Region in the western part of Guinea bordered by the Atlantic Ocean and Sierra Leone. The climate is hot and humid. Coyah Prefecture covers an area of 2,166 square kms and has an estimated population of 264,164 inhabitants, while Forecariah has a population of 244,699 inhabitants distributed in 4,200 square kms in 2014 [7]. As a result of its size and ethnic diversity the region is fairly cosmopolitan in nature. By early December 2014, these two Prefectures had registered 125 cases and 66 deaths with 223 contacts to follow up [6]. Several stakeholders at local, national and international levels were engaged in the fight against the epidemic in these two Prefectures.

**Study design and methods:** a cross-sectional study was conducted in December 2014 using different approaches: (i) Desk review; (ii) Interviews; and (iii) Direct observation.

### Data collection

*Desk review:* available documents at local, national and international levels were studied to better understand the institutional response framework, the stakeholders involved in the response, and the on-going interventions and initiatives.

*Interviews:* interviews were conducted by using a structured guide. A list of stakeholders to be interviewed was established on the basis of available information from the coordination committee and the district health team. In total 30 persons from international agencies and NGOs, Ministry of Health officials at various levels, political leaders, religious and community leaders and community based organisation representatives were interviewed.

*Direct observation:* preparedness of the health facilities, available resources and procedures for prevention and management of EVD were reviewed. Given the urgent nature of the work, the desk review, the interviews and direct observations were carried out simultaneously.

### Data processing and analysis

Both qualitative and quantitative data were collected. Quantitative data were entered and analysed in Excel 2010 and were presented in the form of frequencies or tables. The qualitative data were recorded, grouped, combined and analysed thematically.

**Ethical considerations**: prior to the interviews, appropriate information was given to participants in a language of their choice. All interviewees signed an informed consent form.

## Results

### Coordination mechanisms of the EVD response

**Figure 1:**
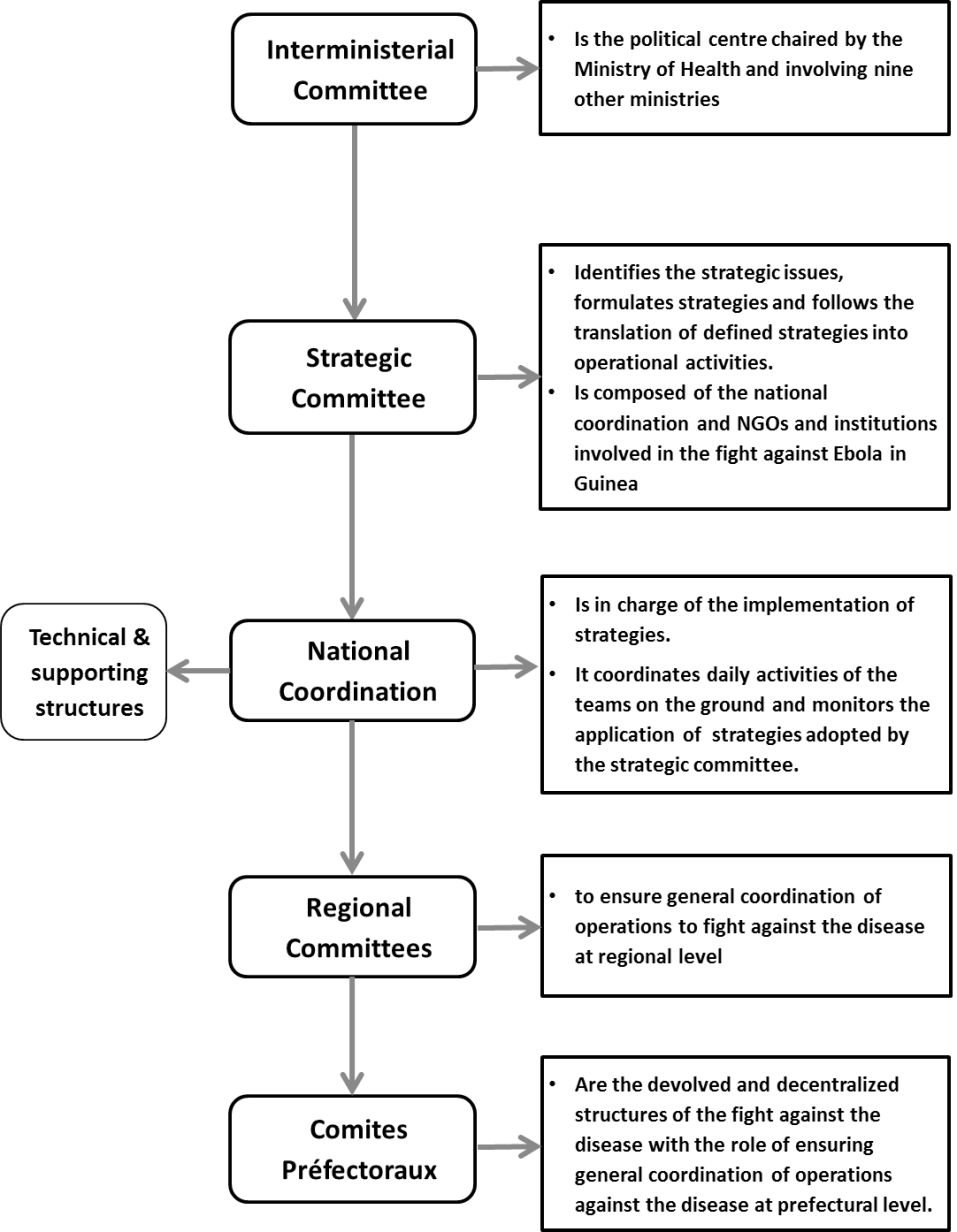
Institutional framework of the fight against Ebola in Guinea

In Guinea, the Ebola outbreak was officially declared in March 2014. The government in collaboration with technical partners prepared a response plan structured around eight strategic intervention points: (i) Establish coordination mechanisms; (ii) Early detection of suspected cases and contacts; (iii) Investigations of suspected cases; (iv) Prompt and effective management of all suspected cases; (v) Management of dead bodies; (vi) Prevention; (vii) Strengthening laboratory services; and (viii) Management of biomedical waste. The response was organized at three levels: central, regional and district, involving various committees (Figure 1).

National coordination meetings are held twice a week, with additional meetings as required. Regional and district committee meetings are convened daily. The Prefecture response Committee, headed by the Prefet (district commissioner) ensures general coordination of interventions at district level. The technical coordination team, led by the prefecture (or district) health director, is in charge of coordinating daily technical activities on the ground.

The prefectoral committee faced many challenges including managing the various daily meetings which were conducted at times that were inconvenient for many health workers and contrary to their wishes. Other challenges included addressing the requests of community-based organizations; managing unwillingness and communities’ resistance; coordinating the multiplicity and diversity of stakeholders, sometimes with conflicting agendas; and managing conflicts between the prefectural coordination committee and the district health teams. Unlike in Coyah where the Prefectural Health Director played the role of Prefectural Director for the fight against Ebola, this structure is led in Forecariah by a coordinator appointed by the national coordination committee

*« The coordinators have been parachuted from the top, without asking for our opinion »* Health personnel, Forecariah.

### Access to diagnostic and treatment services

Health facilities in the two prefectures include two district hospitals, 14 health centres, 57 dispensaries and four private clinics managed by 335 staff. The health workforce mainly comprises nursing aides who represent 42% of the total staff, while physicians are less than 10% of all trained staff.

The health personnel have not been adequately trained on Ebola. One or two staff were identified per facility and invited at the prefecture level to attend training for two or three days on Ebola. Afterward the trained staff was supposed to replicate the training in their respective structures, however no additional budget or material support was provided to facilitate that feedback, and no follow up was made to ensure this took place.

Although the staff of the health centres acknowledged having received enough gloves, they complained that the protective equipment against the Ebola virus (coats/gowns, individual masks, bibs and boots) and sanitation products, in particular, chlorine, were not enough at all peripheral health facilities.

« *We are not ready enough* » Nurse, Forecariah.

The isolation rooms for the Ebola suspected cases in the health facilities were inadequate and might have put the health staff and other patients at risk. The maintenance staff in the two prefectural hospitals and health centres recognised that training was inadequate in infection prevention measures for Ebola.

Health care workers and health managers also confirmed that the epidemic has had a direct impact on priority health programmes with a drastic drop in number of consultations, reduction of the immunization coverage, greater numbers of home deliveries, increase of cases of severe malaria among children and pregnant women, delays in reporting and a decrease in revenue. Logistical issues affected the timely management of suspected cases or dead bodies. Communities and health staff accused organisations in charge of transportation and referral of not responding in time to collect suspected cases.

« *We call them but it takes them three hours or more to come…sometimes even they do not come, and as such we are exposed* » Community Agent, Maferinyah.

If suspected patients reach the transit centre (TC), they are not adequately managed because sometimes it takes up to three days or more before the results of their tests are made available, due to a lack of transport of specimens. A health worker in a TC of Forecariah said:

«*We are told that the PCR takes only 4 hours, but the results of our samples are delayed for days…it is not good to keep patients for a long time in the TC if they do not suffer from Ebola. This is what makes the population angry*».

### Community perception and belief

At the time of the assessment, resistance of communities to Ebola control interventions was reported in nine localities of Coyah and Forecariah. According to local officers, members of CBOs and health staff, communication on Ebola to the populations was a failure from the very beginning and catching up has taken time and this was the authorities’ fault.

« *They (the authorities) said that it was a disease that can't be cured and that kills. Therefore what would the people go to the hospital for?* » Youth leader.

People interviewed acknowledged that social practices related to funerals limited the adoption of safe burial measures in the two prefectures. Furthermore the perception and regard for the authorities and local governance increased their resistance to correct dead body management. Many community members accused the organisation in charge of dead body management of not using proper approaches to change the community practices to avoid or mitigate resistance to safe burials. They were accused of coming into the villages and families masked without prior information or sensitization, and start spraying houses. According to religious leaders, the use of protection masks at the beginning of the epidemic engendered fear in the communities. In addition, the fact that instructions were given from a distance, for example through the media, was seen as something against local traditions. Local officers and CBO members believed that the interventions were interest driven.

« *We are illiterate but we are not stupid. We hear every day that billions of francs are disbursed but we don't receive anything* » Member of a CBO.

### Stigmatization and support to affected individuals and families

The suspected cases, even if they were tested negative afterwards, and the survivors and their families, are strongly stigmatized by the community leading to economic and other hardships.

« *There is a shopkeeper who has been suspected here but finally it was said that it was not Ebola but since then nobody wants to go to his shop*» CBO Member, Forecariah.

At the time of the assessment, there was no sustainable strategy or intervention targeting survivors in the prefectures of Coyah and Forcariah. It was planned to provide financial support to affected families at Forecariah (about 290 USD per family) but this was not carried out as the Coordination Committee feared the reaction that might have resulted if the distribution was selective. A distribution of food at Coyah turned into a riot leading to the theft of the food. Poor identification of beneficiaries, poor planning and distribution, as well as lack of collaboration with security services during the distribution were identified as the main causes of the failure. Two distributions of sanitation and hygiene products at Forecariah were done once without covering the whole prefecture.

### Community involvement and participation

At the onset of the epidemic, the public media, as well as the private media, were widely used by the authorities to inform and advise populations. At the time of the assessment, a new strategy was being put into place consisting of the creation of watchdog committees (WC/VCV) aimed at placing communities in the forefront of the fight against the epidemic. However, many concerns related to community involvement were raised by local officers, religious leaders and other civil society members, underlining the weaknesses in the coordination of interventions including lack of involvement from the beginning in planning and implementing EVD control measures.

«*You are the first NGO that has come to tell us about Ebola and ask us about advice* » Religious leader, Forecariah Centre.

The duplicity of the watchdog committees was also condemned as different institutions created their own watchdog committees in the same areas. The identification process of the members of these watchdog committees was criticized. Interventions of the institutions and NGOs within the communities were not harmonized leading to duplications for instance in the distribution of sanitation products and food.

« If you put ethnic groups and politics into the choice of your collaborators for the fight against Ebola, where are we going to get to? » Religious leader, Forecariah Centre.

Another weakness was the use of foreigners for awareness raising in the community, which was seen as a real invasion.

«*They take people from other places and whom we are not familiar with, to come and tell us stories, why can't they take us to talk to our relatives here?* » Young from, Coyah.

Behind these denials, there were also hidden economic constraints within populations as a result of the epidemic. Not using the unemployed local manpower was taken very badly by communities.

« *You come to convince someone who has spent a whole day without eating. Do you think you can succeed in doing so?* » Religious leader, Coyah

The strategy used for funerals was severely criticized, the major criticism being that they did not receive prior information or sensitization. This engendered frustration within families of Ebola victims during funerals and led to misunderstandings of protocols and poor management of dead bodies.

« *You remain here and you see them coming in, to spray ….with what?…and what if they are the ones spreading the virus?* » CBO Member, Forecariah.

Local officials witnessed that communication issues hindered efforts to accelerate the control of the epidemic.

« *One of the major causes of the spreading of the epidemic is, indeed, the reluctance of the population at the forefront of the intervention response and this can only be sustainably solved by communication*» Member of National Coordination Committee.

## Discussion

This is to our knowledge one of the first studies reporting on the Ebola virus disease preparedness and the perceptions of stakeholders in Guinea. Overall, the two districts were still inadequately prepared to control the EVD outbreak almost one year since its onset. The same observation has been reported in previous similar assessments from Liberia and Sierra Leone, two of the three most affected countries [8, 9]. This confirms the fact that the Ebola outbreak happened in a context where health systems were already fragile with a very limited capacity to respond to a rapid epidemic [10, 11].

The study has some limitations. First of all it was a rapid assessment in an emergency situation; this did not allow appropriate sampling and time for in-depth interviews as well focus group discussions. Secondly, the assessment was conducted in an evolving and changing environment where Ebola case numbers and field interventions were continuously changing. However, the study was well perceived by communities and stakeholders who generally responded positively to interviews.

EVD outbreaks require a rapid and structured response. A clearly defined chain of command and organizational structure, effective resource management, and advanced planning and coordination are critical aspects of the response [12]. We demonstrated that because the health district teams were left out, the prefectoral coordination committees lacked authority and leadership to adequately manage EVD control activities. In addition, the lack of adequate training of health care workers, especially in infection prevention measures and the shortage of equipment and supplies to implement these measures delayed an appropriate response [8, 13]. In Sierra Leone, a rapid needs assessment revealed inadequate training of health care workers in infection prevention measures and lack of standard operating procedures in health facilities [8]. Furthermore health care workers themselves have been severely affected by the disease and some of them had left their positions because of the fear of being infected [14, 15]. This situation added to the fear and distrust of communities towards health services which could explain the drastic reduction in the use of priority health programs (maternal, neonatal and child health) in the two districts [16].

Long delays in Ebola patient transportation and late reporting of laboratory results probably contributed to the increased case fatality rate, thus reinforcing the fear within communities. In Sierra Leone, laboratory sample result turnaround time varied and sometimes took as long as one week for areas that were distant from Ebola diagnostic laboratories [8]. In Liberia, because of shortage of ambulances, two days were required to transport patients to the treatment center in Monrovia [17]. In another report, investigation teams reported walking for up to 8 hours in the rainy season to reach communities where cases had been reported [18]. Such situations create distrust, fear and panic.

Distrust has played a critical role in the challenges in controlling the outbreak, largely in relation to community perceptions, beliefs and views. Inadequate information generates distrust which leads to fear, panic and ultimately to violence [19].

Misconceptions around the disease, ignorance of local practices and cultural approaches, and the lack of local communities’ participation and involvement increased the reluctance and resistance of the communities in the two health districts [20]. The two districts remained among the localities that continued to report reluctance in the country and as of March 1, 2015, only these two districts and the Capital city Conakry were reporting daily new cases of Ebola in Guinea [21]. Because the EVD control required changes in social and cultural practices such as hand greeting, burial practices and even simple visits to a sick relative, ignoring local culturally accepted approaches at the beginning of the epidemic was perceived as a lack of respect to communities from the authorities and other stakeholders. In Liberia and Sierra Leone, misconceptions and erroneous beliefs such as medical staff being paid for each patient referred, patients being injected by health care workers with Ebola, foreigners bringing Ebola to areas, or blood of patients being taken for financial gain or magical power were reported and identified as challenges that needed to be addressed adequately [8, 9, 20, 22].

Finally in the context of poverty worsened by the outbreak, communities were expecting greater solidarity from authorities, institutions and NGOs intervening in the field, in particular food distribution as well as employment of local populations, but most importantly listening to and involving them [23, 24].

## Conclusion

In Guinea, the weak health system, inadequate preparedness and response to EVD, poor coordination, inappropriate information, misconceptions and lack of dialogue in the context of poor socioeconomic development, created a gap between the population and the government and its partners, which has led to distrust and to the disastrous spread of the epidemic.

In rural areas, EVD control requires rapid response including prompt transport and management of patients, community surveillance and safe burial practices. The response needs to be supported by an effective decentralized coordination mechanism involving local communities at various levels. Communication needs to be tailored to the local context and much attention given to the implications of the underlying message.

There is a need to rethink the process and flow of control interventions in the context of EVD that involve communities and understand their views, concerns and fears in order to work side by side with them to define and implement appropriate control strategies that respect local customs, beliefs and individuals.

## References

[1] World Health Organisation (2015). One Year into the Ebola Epidemic: a deadly, tenacious and unforgiving virus. Ebola Virus Disease.

[2] Agyepong Irene A (2015). A Systems View and Lessons from the Ongoing Ebola Virus Disease Outbreak in West Africa. Ghana Medical Journal..

[3] World Health Organisation (2014). WHO, Ebola Response Roadmap Situation Report, 3 December 2014. WHO, Ebola Virus Disease. (Online).

[4] UNICEF (2015). Ebola Situation report. reliefweb.

[5] Remco van de Pas (2015). On poilitics and pathogen: why does community resistance persit in Guinea.

[6] Coordination Nationale Ebola, Guinee and OMS (2014). Rapport de la Situation Epidémiologique, Maladie a Virus Ebola en Guinée du 03 Decembre 2014.

[7] Institut National des Statistiques de Guinee (INS) (2015). La Guinée en bref.

[8] Pathmanathan Ishani, O'Connor Katherine A, Adams Monica L (2014). Rapid assessment of Ebola infection prevention and control needs-six districts, Sierra Leone, October 2014. MMWR Morb Mortal Wkly Rep..

[9] Dynes Michelle M, Miller Laura, Sam Tamba, Vandi Mohamed Alex, Tomczyk Barbara (2015). Perceptions of the risk for Ebola and health facility use among health workers and pregnant and lactating women-Kenema District, Sierra Leone, September 2014. MMWR Morb Mortal Wkly Rep..

[10] Piot Peter, Muyembe Jean Jacques, Edmunds Wjohns (2014). Ebola in west Africa: from disease outbreak to humanitarian crisis. Lancet Infect Dis..

[11] Kieny Marie-Paule, Evans David B, Schmets Gerard, Kadandale Sowmya (2014). Health-system resilience: reflections on the Ebola crisis in western Africa. Bull World Health Organ..

[12] Centers for Disease Control and Prevention. Guinea Interministerial Committee for Response Against the Ebola Virus, World Health Organization, CDC Guinea Response Team (2015). Update: ebola virus disease epidemic - west Africa, february 2015. MMWR Morb Mortal Wkly Rep.

[13] Osterholm Michael T, Moore Kristine A, Gostin Lawrence O (2014). Public Health in the Age of Ebola in West Africa. JAMA Intern Med..

[14] Delamou Alexandre, Beavogui Abdoul Habib, Konde Mandy Kader, van Griensven Johan, De Brouwere Vincent (2015). Ebola: better protection needed for Guinean health-care workers. Lancet..

[15] Coordination Nationale Ebola, Guinee and OMS (2014). Rapport de la Situation Epidémiologique, Maladie a Virus Ebola en Guinée du 31 Decembre 2014.

[16] Delamou Alexandre, Hammonds Rachel, Caluwaerts Severine, Utz Bettina, Delvaux Therese (2014). Ebola in Africa: beyond epidemics, reproductive health in crisis. Lancet..

[17] Nyenswah Tolbert, Blackley David J, Freeman Tabeh, Lindblade Kim A (2015). Community quarantine to interrupt ebola virus transmission - mawah village, bong county, liberia, august-october, 2014. MMWR Morb Mortal Wkly Rep..

[18] Summers Aimee, Nyenswah Tolbert G, Montgomery Joel M, Neatherlin John, Tappero Jordan W (2014). Challenges in responding to the ebola epidemic - four rural counties, Liberia, August-November 2014. MMWR Morb Mortal Wkly Rep..

[19] Sharfstein Joshua M (2015). On fear, distrust, and Ebola. JAMA.

[20] Nielsen Carrie F, Kidd Sarah, Sillah Ansumana RM, Davis Edward, Mermin Jonathan, Kilmarx Peter H (2015). Improving burial practices and cemetery management during an Ebola virus disease epidemic - Sierra Leone, 2014. MMWR Morb Mortal Wkly Rep..

[21] Coordination Nationale Ebola, Guinee and OMS (2015). Rapport de la Situation Epidémiologique, Maladie a Virus Ebola en Guinée du 1er Mars 2015.

[22] Bannister-Tyrrell Melanie, Gryseel Charlotte, Delamou Alexandre, D'Alessandro Umberto, van Griensven Johan, Grietens Koen Peeters, the Ebola_Tx Trial Platform (2015). Blood as medicine: social meanings of blood and the success of Ebola trials. Lancet..

[23] The Lancet (2014). Ebola: protection of health-care workers (Editorial). Lancet.

[24] Hagan Jose E, Smith Wilmot, Pillai Satish K, Yeoman Kristin, Gupta Sundeep (2015). Implementation of ebola case-finding using a village chieftaincy taskforce in a remote outbreak - liberia, 2014. MMWR Morb Mortal Wkly Rep..

